# UPLC-MS based integrated plasma proteomic and metabolomic profiling of TSC-RAML and its relationship with everolimus treatment

**DOI:** 10.3389/fmolb.2023.1000248

**Published:** 2023-02-20

**Authors:** Zhan Wang, Xiaoyan Liu, Wenda Wang, Jiyu Xu, Haidan Sun, Jing Wei, Yuncui Yu, Yang Zhao, Xu Wang, Zhangcheng Liao, Wei Sun, Lulu Jia, Yushi Zhang

**Affiliations:** ^1^ Department of Urology, Peking Union Medical College Hospital, Chinese Academy of Medical Science and Peking Union Medical College, Beijing, China; ^2^ School of Basic Medical College, Core facility of instrument, Institution of Basic Medical Sciences, Chinese Academy of Medical Sciences, Beijing, China; ^3^ Clinical Research Center, National Center for Children’s Health, Beijing Children’s Hospital, Capital Medical University, Beijing, China

**Keywords:** UPLC-MS, proteomics, metabolomics, tuberous sclerosis complex, everolimus

## Abstract

**Aim:** To profile the plasma proteomics and metabolomics of patients with renal cysts, sporadic angiomyolipoma (S-AML) and tuberous sclerosis complex related angiomyolipoma (TSC-RAML) before and after everolimus treatment, and to find potential diagnostic and prognostic biomarkers as well as reveal the underlying mechanism of TSC tumorigenesis.

**Materials and Methods:** We retrospectively measured the plasma proteins and metabolites from November 2016 to November 2017 in a cohort of pre-treatment and post-treatment TSC-RAML patients and compared them with renal cyst and S-AML patients by ultra-performance liquid chromatography-mass spectrometer (UPLC-MS). The tumor reduction rates of TSC-RAML were assessed and correlated with the plasma protein and metabolite levels. In addition, functional analysis based on differentially expressed molecules was performed to reveal the underlying mechanisms.

**Results:** Eighty-five patients with one hundred and ten plasma samples were enrolled in our study. Multiple proteins and metabolites, such as pre-melanosome protein (PMEL) and S-adenosylmethionine (SAM), demonstrated both diagnostic and prognostic effects. Functional analysis revealed many dysregulated pathways, including angiogenesis synthesis, smooth muscle proliferation and migration, amino acid metabolism and glycerophospholipid metabolism.

**Conclusion:** The plasma proteomics and metabolomics pattern of TSC-RAML was clearly different from that of other renal tumors, and the differentially expressed plasma molecules could be used as prognostic and diagnostic biomarkers. The dysregulated pathways, such as angiogenesis and amino acid metabolism, may shed new light on the treatment of TSC-RAML.

## Background

Tuberous sclerosis complex (TSC) is a rare disease caused by germline mutations of tumor suppressor genes in either the *TSC1* gene on chromosome 9 or the *TSC2* gene on chromosome 16 (Henske et al., 2016). Its incidence is approximately 1 in 6,000–10,000, and there are around 2 million patients worldwide, although the rate may be greatly underestimated due to large numbers of undiagnosed patients (Lam et al., 2018). TSC threatens multiple organs throughout the body and causes corresponding distinctive manifestations, including subependymal giant cell astrocytoma (SEGA) in the brain, rhabdomyosarcoma (RA) in the heart, lymphangioleiomyomatosis (LAM) in the lung, angiomyolipoma (AML) in the kidney and so on.

For the underlying mechanism, the most widely acknowledged theory is that silencing of the TSC complex caused by mutations could lead to overactivation of the mammalian target of rapamycin (mTOR) signaling pathway, which has been proven to be critical in various physiological processes, such as regulating cell growth, metabolism and autophagy (Yang et al., 2013; Ranek et al., 2019). Aberrant constitutive mTOR pathway activation could result in unregulated cell proliferation, migration, and invasion and finally cause hamartoma in different organs (Bottolo et al., 2020). Based on the above mechanism, mTOR inhibitors, including rapamycin and everolimus, have been developed to control the various manifestations, including renal AML (Bissler et al., 2013; Cai et al., 2018), brain SEGA (Krueger et al., 2010; Franz et al., 2013) and pulmonary LAM (McCormack et al., 2011).

As the most common cause of early death among patients with TSC (Shepherd et al., 1991; Amin et al., 2017), the renal lesions have three main forms, namely, AML (the most common, making up more than 80%), renal cysts and renal cell carcinoma. The abrupt rupture of TSC related AML (TSC-RAML) is a common cause of mortality and is sometimes referred to as a “ticking bomb” within the body. Exist-2 is thus far the largest multi-center randomized controlled trial assessing the effect of everolimus on TSC-RAML (Bissler et al., 2013). This trial validated its efficacy with a 42% response rate and an acceptable safety profile, making everolimus the only drug approved by the Food and Drug Administration of America to treat TSC-RAML. Our center has also conducted a 2-year, nonrandomized, open-label, phase 2 clinical trial, and the result showed that 50% volume reduction rate reached 52.94% at 3 months and 58.82% at 6 months, further confirming the favorable effect of mTOR inhibitors on TSC-RAML (Cai et al., 2018).

In the EXIST-2 trial, plasma VEGF-D and collagen IV levels were found to be potential prognostic as well as diagnostic biomarkers, and these results have been validated by subsequent studies not only in TSC-RAML (Dabora et al., 2011; Malinowska et al., 2013) but also in the TSC-LAM (Young et al., 2010; Xu et al., 2013; Amaral et al., 2019). So far, very few efficient biomarkers have been discovered to guide clinical treatment or follow-up of patients with TSC.

mTOR is an atypical serine/threonine protein kinase that forms two distinct signaling complexes, mTORC1 and mTORC2, which are distinguished primarily by their association with Raptor or Rictor, respectively (Martin et al., 2014). Through direct phosphorylation and activation of S6 kinase 1 (S6K) and inactivation of 4E-BP1, mTORC1 regulates many cellular metabolisms, such as amino acid, glucose, nucleotide, fatty acid and lipid metabolism (Morita et al., 2015; Mossmann et al., 2018). During this process, massive proteomic and metabolomic hallmarks will be produced if mTOR continuously activated. As one of the most commonly used high-throughput approaches to detect proteome and metabolome in biofluids, ultra-performance liquid chromatography-mass spectrometer (UPLC-MS) has been widely applied in searching for candidate diagnostic and prognostic biomarkers and potential drug targets (Wang et al., 2019; Blomme et al., 2020; Sovio et al., 2020; Behsaz et al., 2021; de la Calle Arregui et al., 2021; Wang C. Y. et al., 2021; Wang Z. et al., 2021).

Therefore, the aim of our study was to retrospectively analyze the plasma proteomic and metabolomic profiles with UPLC-MS and to search for diagnostic and prognostic markers of TSC-RAML to guide clinical management.

## Materials and methods

### Human samples and clinical data

This study was conducted at Peking Union Medical College Hospital from November 2016 to November 2017 and was approved by the Institutional Review Board of Peking Union Medical College Hospital and the Institute of Basic Medical Sciences, Chinese Academy of Medical Sciences (Approval number: KS2020127). This research was carried out according to the Code of Ethics of the World Medical Association (Declaration of Helsinki), and formally written consent documents were provided by every participant before been enrolled in this study.

The inclusion criteria were as follows: 1) The TSC patients met the clinical or genetic diagnosis of TSC according to the International Tuberous Sclerosis Complex Consensus Conference in 2012 and took oral everolimus at a dose of 10 mg/qd for at least 6 months. 2) The S-AML patients received partial nephrectomy and the diagnosis was confirmed as AML. 3) All plasma samples from the TSC patients were collected pre-treatment and 3 or 6 months after initiating everolimus treatment. 4) Patients with renal cysts were considered healthy controls, and blood samples were collected preoperatively during the same period.

The exclusion criteria were: 1) Those who had no pre-operative or pre-treatment plasma in our sample bank. 2) Patients who had malignant tumors or metabolomic diseases such as diabetes and hyperlipidemia.

All the enrolled TSC patients were assessed independently by two radiologists to determine the tumor volume at baseline, every 3 months within the first year, every 6 months within the second year and yearly thereafter. The maximum AML volume was used to calculate the tumor response, and >50% volume reduction was regarded as effective. All TSC patients received next-generation gene sequencing (NGS) to assess their *TSC* gene mutations. The exact process of NGS were described in our previously published article (Wang et al., 2022a).

### Sample collection

Whole blood samples were collected in the morning at 07:00 am–09:00 am with at least 10 h of fasting to eliminate the impact of diet. The 4 mL EDTA tubes with whole blood were transferred and separated by density gradient centrifugation within 1 h after collection. The plasma was stored at −80°C until conducting the formal experiment.

### Sample preparation for proteomics

To remove the highly abundant proteins (including albumin, IgA and IgD) from the plasma, High Select™ Top14 Abundant Protein Depletion Mini Spin Columns (Thermo Fisher Scientific, MA. United States) were applied according to the manufacturer’s instructions (as attached in Supplementary Material S1). After this procedure, we obtained 300 µL samples with the highly abundant proteins removed. Ten microliters of each sample were removed to measure the protein concentration by the BCA assay (Pierce).

Every 100 mg of protein was reduced with 20 mM dithiothreitol (DTT) for 5 min at 95°C and subsequently alkylated with 50 mM iodoacetamide for 45 min at room temperature in the dark. Protein digestion was carried out using the filter-aided sample preparation technique (FASP). Proteins were loaded onto 30 kDa filter devices (Pall, Port Washington, NY, United States). Trypsin (Trypsin Gold, mass spec grade, Promega, WI, United States) was added (enzyme to protein ratio of 1:50), and the samples were incubated at 37°C overnight. The samples were centrifuged at ×14,000 g, and approximately 30 µL of the liquid was used for analysis.

For the quality control (QC) samples, 3 µL was taken from 23 randomly selected representative samples and mixed with the testing samples together, and the mixture was loaded with the testing samples. The QC samples were injected every 10 samples. All the samples were loaded on the autosamplers with a mixture of iRT.

### ESI-LC-MS/MS for proteome library generation

The pooled peptide samples of each group were separated by high-pH RPLC columns (4.6 mm × 250 mm, C18, 3 μm; Waters, United States). Each pooled sample was loaded onto the column in buffer A1 (H2O, pH 10). The elution gradient was 5%–30% buffer B1 (90% ACN, pH 10; flow rate, 1 mL/min) for 30 min. The eluted peptides were collected at one fraction per minute. After lyophilization, the 30 fractions were resuspended in 0.1% formic acid and then concatenated into 10 fractions by combining fractions 1, 11, 21, and so on. To generate the spectral library, the fractions from RPLC were analyzed in DDA mode. The parameters were set as follows: the MS was recorded at 350–1,500 m/z at a resolution of 60,000 m/z; the maximum injection time was 50 ms, the auto gain control (AGC) was 1e6, and the cycle time was 3 s. MS/MS scans were performed at a resolution of 15,000 with an isolation window of 1.6 Da and a collision energy at 32% (HCD); the AGC target was 50,000, and the maximum injection time was 30 ms.

### ESI-LC-MS/MS for proteome data-independent acquisition analysis

The digested peptides were dissolved in 0.1% formic acid and separated on an RP C18 self-packing capillary LC column (75 μm × 150 mm, 3 μm). The elution gradient was 5%–30% buffer B2 (0.1% formic acid, 99.9% ACN; flow rate, 0.3 μL/min) for 60 min. For MS acquisition, the variable isolation window DIA method with 38 windows was developed. The specific window lists were constructed based on the DDA experiment of the pooled sample. The full scan was set at a resolution of 120,000 over the m/z range of 400 to 900, followed by DIA scans with a resolution of 30,000; the HCD collision energy was 32%, the AGC target was 1E6, and the maximal injection time was 50 ms.

### Spectral library generation

To generate a comprehensive spectral library, the pooled sample from each group was processed. The DDA data were processed using Proteome Discoverer (Thermo Scientific, Germany) software and searched against the human SwissProt database appended with the iRT fusion protein sequence (Biognosys). A maximum of two missed cleavages for trypsin was used, cysteine carbamidomethylation was set as a fixed modification, and methionine oxidation deamination and +43 on Kn (carbamyl) were used as variable modifications. The parent and fragment ion mass tolerances were set to 10 ppm and 0.02 Da, respectively. The applied false discovery rate (FDR) cutoff was 0.01 at the protein level. The results were then imported into Spectronaut Pulsar (Biognosys, Switzerland) software to generate the library. Additionally, DIA data were imported into Spectronaut Pulsar software and searched against the human SwissProt database to generate the DIA library. The final library was generated by combining the DDA and DIA libraries of all the enrolled samples.

### Data analysis

The DIA-MS data were analyzed using the Spectronaut Pulsar (Biognosys, Switzerland) with the default settings. All of the results were filtered with a Q-value cutoff of 0.01 (corresponding to an FDR of 1%). Proteins identified in more than 50% of the samples in at least one subgroup were retained for further analysis. Missing values were imputed based on the k-nearest neighbor method or by the minimum value (details provided in Supplementary Figure S1A).

Raw proteomics data were log10 transformed and then centralized. Student’s t-test was used, and the software was R (version 4.1.1). Any differential proteins that fulfilled all of the limitations were considered significant: 1) *p*-value <0.05; and 2) Fold change ≥2.

### Sample preparation for metabolomics

First, each mixture of plasma sample (50 μL) and H2O (150 µL) was vortexed for 30 s. Then, 400 µL acetonitrile was added to the mixture, vortexed for another 30 s and centrifuged at ×14,000 g for 10 min. The samples were dried under vacuum, and the supernatant was then blended with 200 μL of 2% acetonitrile. Before being transferred to the autosamplers, 10 kDa molecular weight cutoff ultracentrifugation filters (Millipore Amicon Ultra, MA) were applied to separate the blood metabolites from the larger molecules. QC samples were prepared by mixing aliquots of one hundred and ten representative samples and they were injected every ten samples throughout the analytical run to assess the method stability and repeatability.

### UPLC-MS analysis for metabolomics

The Waters ACQUITY H-class LC system coupled with an AB Sciex TripleTOF 5600 (AB Sciex, United States) was launched to perform the ultra-performance LC-MS analyses of the plasma samples. We separated the plasma metabolites with a 17 min gradient on a Waters HSS C18 column (3.0 × 100 mm, 1.7 μm), and the flow speed was 0.5 mL/min. Mobile phases A and B were 0.1% formic acid in H2O and acetonitrile, respectively. The gradient was as follows: 0–1 min, 2% solvent B; 1–3 min, 2%–15% solvent B; 3–6 min, 15%–50% solvent B; 6–9 min, 50%–95% solvent B; 9–9.1 min, 95%–100% solvent B; 9.1–12 min, 100% solvent B; 12–12.1 min, 100%–2% solvent B; and 12–17 min, 2% solvent B. The column temperature was 45°C. Data dependent acquisition mode was used to acquire the MS and MS/MS spectra. The 10 most abundant ions were submitted for MS/MS fragmentation with a collision energy of 35+-15 eV.

### Data processing for metabolomics

Progenesis QI (Waters, Milford, MA, United States) software was applied to analyze the raw data. The data handling and metabolite identification processes can be found in the Supplementary Materials S2. The exported results file consisting of m/z, retention time and relative peak intensity was submitted for further statistical analysis. We established various statistical techniques, such as missing value estimation, log10 transformation and Z score scaling; thus, the features could be more comparable in MetaAnalyst 5.0. The data handling process is depicted in Supplementary Figure S2B, similar to the proteomic process. Any differential variables that fulfilled all the limitations were considered significant: 1) *p*-value <0.05; and 2) Fold change ≥1.5.

### Functional enrichment analysis

The R package “ClusterProfiler” was applied to conduct Gene Oncology (GO) enrichment analysis (Yu et al., 2012). The interaction network between the proteomics and metabolomics and the functional enrichment of the differential metabolites used MetaboAnalyst 5.0 (http://www.metabo analyst.ca). The “WGCNA” package was applied to find characteristic markers of every group (Langfelder and Horvath, 2008; Langfelder and Horvath, 2012). GSEA application (version 4.1.0) was applied to perform GSEA hallmark analysis. The “ClueGo” module of Cytoscape (version 3.9.0, United States) was launched to conduct and display the functional enrichment results (Shannon et al., 2003).

### Statistical analysis

Unless specially mentioned above, R (version 4.1.1) was used to perform all the analyzes and construct all the figures. All above tests were two-sided and *p*-value ≤ 0.05 was regarded as statistically significant. The R package “pwr” (version 1.3–0) has already been applied to calculate the minimum samples required for the analysis.

## Results

### Human samples and clinical data

**FIGURE 1:**
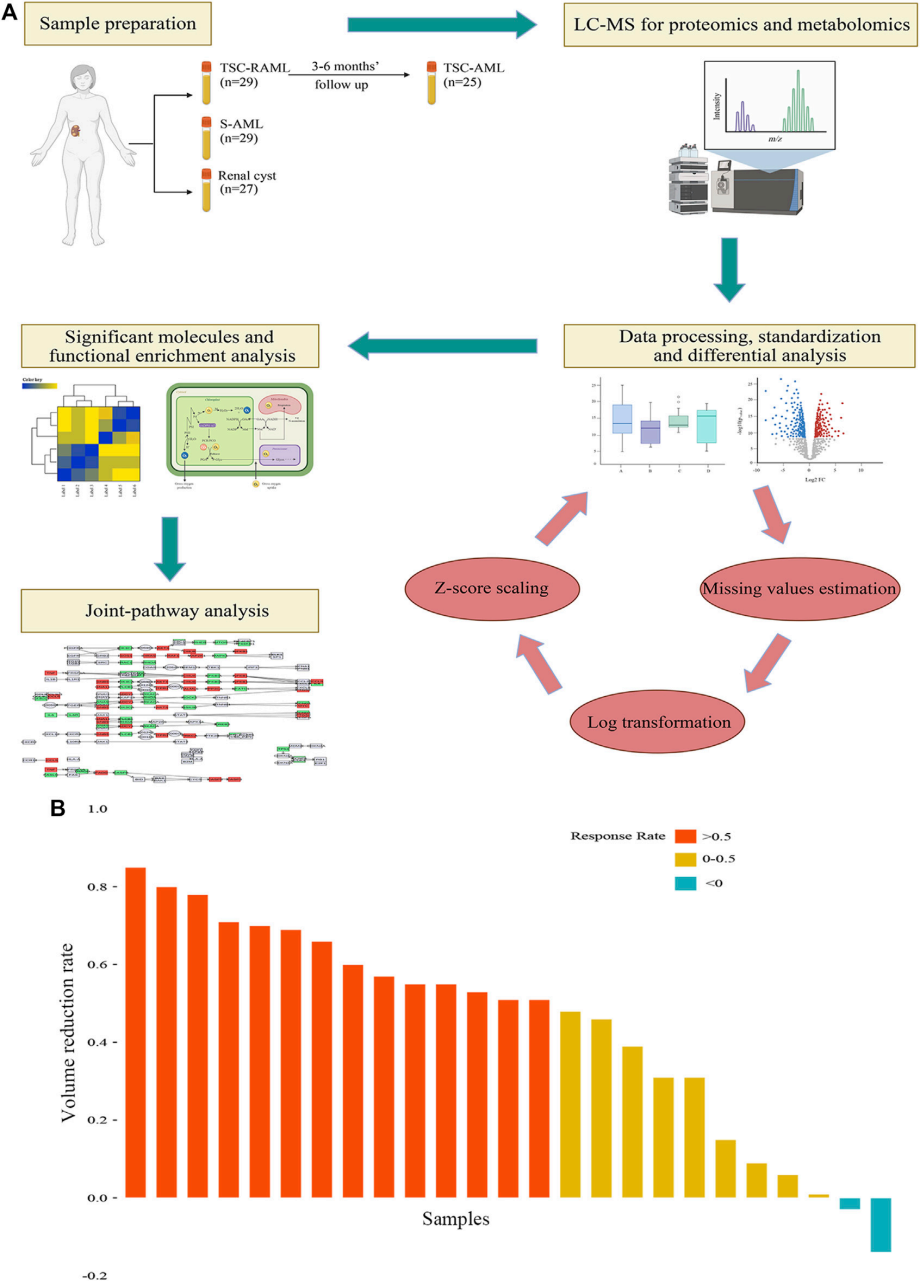
**(A)** The workflow of our study. **(B)** The tumor volume reduction rate of TSC-RAML after everolimus treatment.

A total of 85 patients were enrolled in our final analysis, including 29 TSC-RAML, 29 S-AML and 27 renal cyst (CY) patients. Among the 29 TSC-RAML patients, 25 had double samples, namely, the pre-treatment (pre_TSC) and post-treatment plasma (post_TSC) samples. The basic clinical information of all enrolled patients is shown in Table 1, and the workflow of this study is depicted in Figure 1.

**TABLE 1:** The baseline information of all enrolled patients.

Items	TSC-RAML		S-AML	Renal cyst
	Pre_treatment	Post_treatment		
Cases (n)	29	25	29	27
Age (years)	29	29.5	39	47
—	(14, 42)	(18, 42)	(15, 54)	(13,78)
Gender (M/F)	11/18	9/16	5/24	13/14

In terms of mutations in the 29 TSC patients, 9 had nonsense mutations, 6 had shift frame mutations, 6 did not have any mutations detected, 4 had missense mutations and 4 had other mutations (2 with base deletions, 1 with an insertion and 1 splicing variation), which can be seen in Supplementary Table S1.

Regarding the treatment effect of everolimus, the results showed that after 3–6 months of treatment, 92% (23/25) of patients experienced tumor reduction, and more than half (56%, 14/25) of patients reached the endpoint of 50% tumor reduction (as depicted in Figure 1B).

### The proteome of TSC-RAML, S-AML and renal cyst

Quantitative proteomic data of one hundred and ten plasma samples based on the DIA mode were created. After processing the raw data, a total of 997 proteins remained for further analysis (the process can be seen in Supplementary Figure S1A).

**FIGURE 2:**
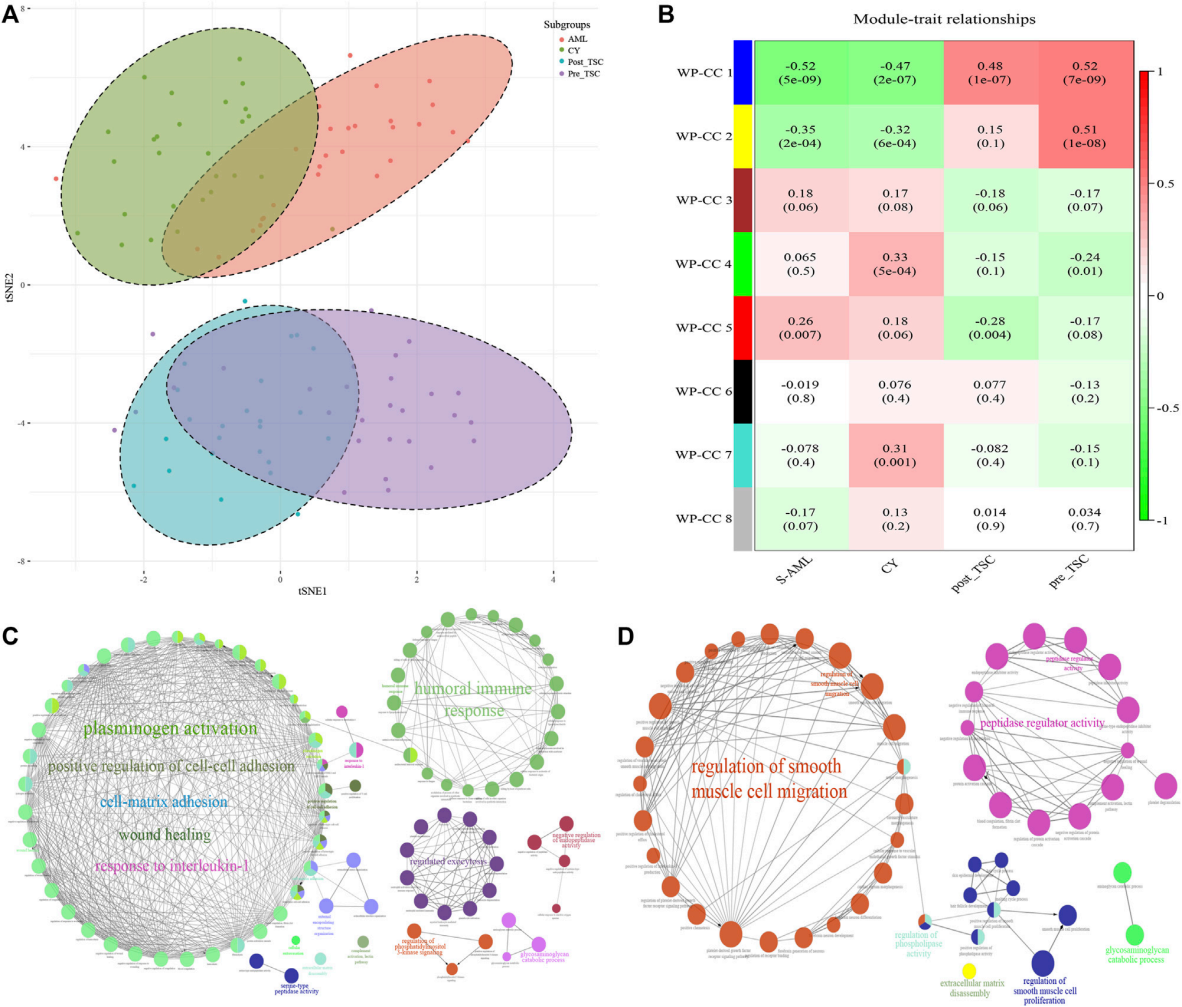
Proteomics profile of all enrolled patients. **(A)** t-SNE analysis revealed the unique proteome of TSC-RAML. **(B)** WGCNA identified 8 whole proteome co-expression clusters (WP-CC). **(C)** Functional analysis of the “WP-CC 1” cluster showed dysregulated cell-matrix adhesion. **(D)** Functional analysis of the “WP-CC 2” cluster showed altered regulation of smooth muscle cell migration.

First, t-distributed stochastic neighbor embedding (t-SNE) was applied, and distinctions within the subgroups could be observed, although there was some overlap within the S-AML vs. the CY and post_TSC vs. pre_TSC (Figure 2A). Then, we performed gene co-expression clustering, pathway analysis and functional module classification by means of weighted gene correlation network analysis (WGCNA) and “ClueGO”. All 997 proteins were classified into eight whole proteome coexpression clusters (WP-CC), among which the “WP-CC 1” module was positively and significantly associated with TSC-RAML but negatively associated with renal cysts and S-AML (Figure 2B). In addition, the cluster of “WP-CC 2” demonstrated the same tendency. The proteins within the two clusters were then enrolled into the functional analysis and displayed by the “ClueGO”. Interestingly, the proteins in the two rewired clusters were mainly enriched in the glycosaminoglycan catabolic process, regulation of phosphatidylinositol 3-kinase signaling and cell-matrix adhesion (WP-CC 1, Figure 2C) and glycosaminoglycan catabolic process, regulation of smooth muscle cell migration and proliferation, extracellular matrix disassembly, and regulation of phospholipase activity pathways (WP-CC 2, Figure 2D).

### Comparison of the proteomes of TSC-RAML, S-AML and renal cysts

**FIGURE 3:**
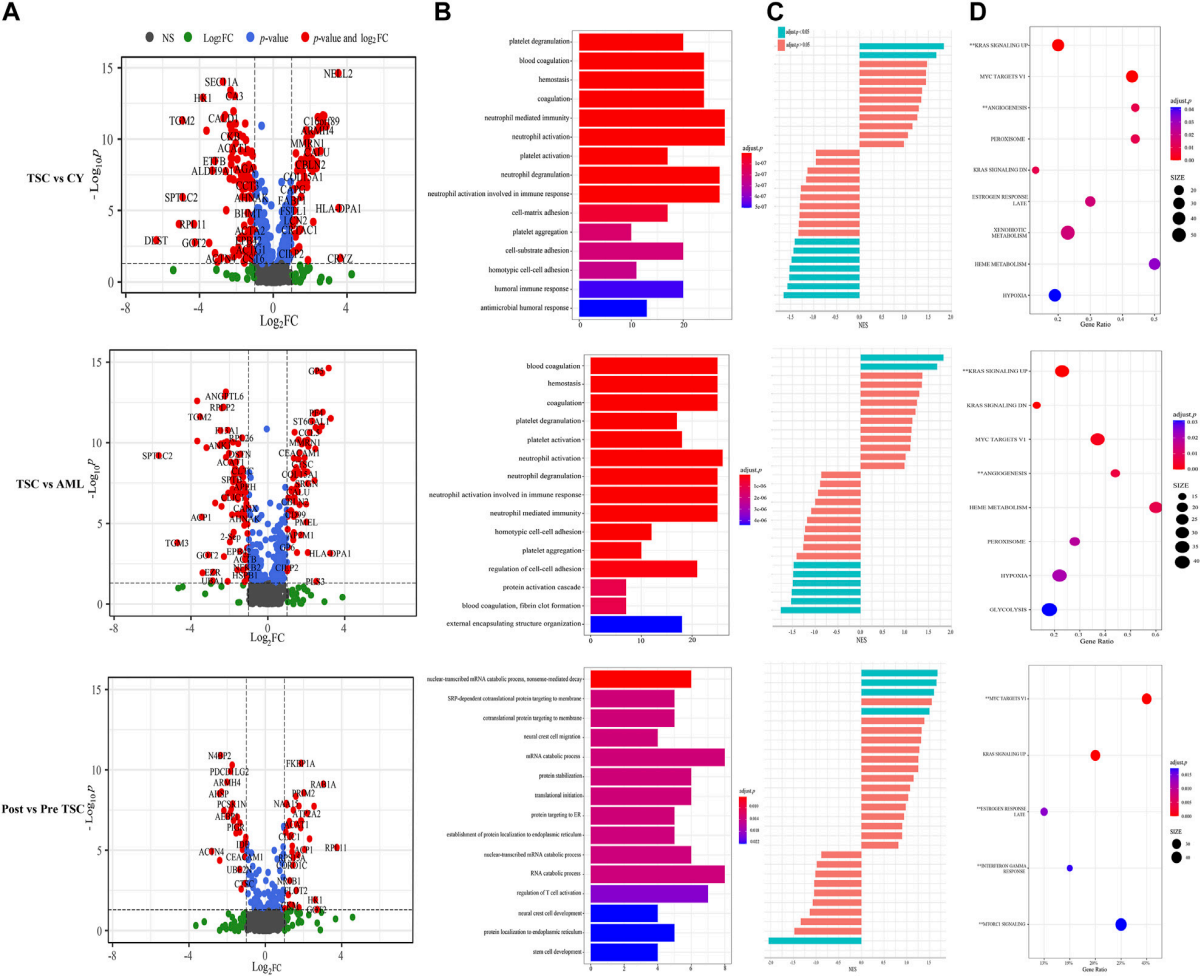
Comparison within different groups and functional analysis of DE proteins. **(A)** Volcano plot based on the threshold. **(B)** GO enrichment of DE proteins. **(C)** GSEA items of the proteomics. **(D)** GSEA enrichment of all of the statistically significant items (**significantly upregulated; without annotation: significantly downregulated).

According to the threshold (FC ≥ 2, *p* ≤ 0.05), there were 198 differentially expressed (DE) proteins in the pre-treatment TSC-RAML group compared with the renal cyst group, including 73 upregulated and 125 downregulated molecules (Figure 3A, above). Gene oncology (GO) functional enrichment revealed that there were several dysregulated pathways, including platelet degranulation, blood coagulation, hemostasis, cell-matrix adhesion and humoral immune response within the two groups (Figure 3B, above). Since the GO functional enrichment of DE proteins may neglect pivotal information regarding the interactive mechanism, we additionally applied gene set enrichment analysis (GSEA) regarding hallmarks with the molecular signature database (MSigDB v7.4). The results of GSEA hallmark analysis demonstrated that compared with the renal cyst group, the pre-treatment TSC-RAML group possessed two significantly upregulated and seven significantly downregulated pathways (Figures 3C, D). As expected, the angiogenesis pathway was significantly upregulated in the plasma of TSC-RAML patients, which was in accordance with the pathological process of angiomyolipoma biosynthesis (Xian et al., 2011). In addition, KRAS signaling up was upregulation.

Due to the characteristic symptoms and specific mutations of the TSC1 or TSC2 genes, TSC-RAML is quite different from S-AML in many aspects, including multifocal, a larger tumor volume and a higher incidence of tumor rupture, which is the main cause of death among adult TSC-RAML patients (Amin et al., 2017; Lam et al., 2018). Therefore, we also analyzed the plasma proteins in TSC-RAML and S-AML to illustrate their differences at the proteome level.

According to the differential analysis, we identified 174 DE proteins, namely, 77 upregulated and 97 downregulated proteins (Figure 3A, middle). Similarly, the GO enrichment analysis of all DE proteins suggested dysregulated blood coagulation, hemostasis, etc., (Figure 3B, middle). Furthermore, the hallmark GSEA showed that compared with S-AML, TSC-RAML had high targets of angiogenesis and the K-RAS signaling up pathway, which was quite similar to the results of TSC vs. renal cysts (Figures 3C, D).

**FIGURE 4:**
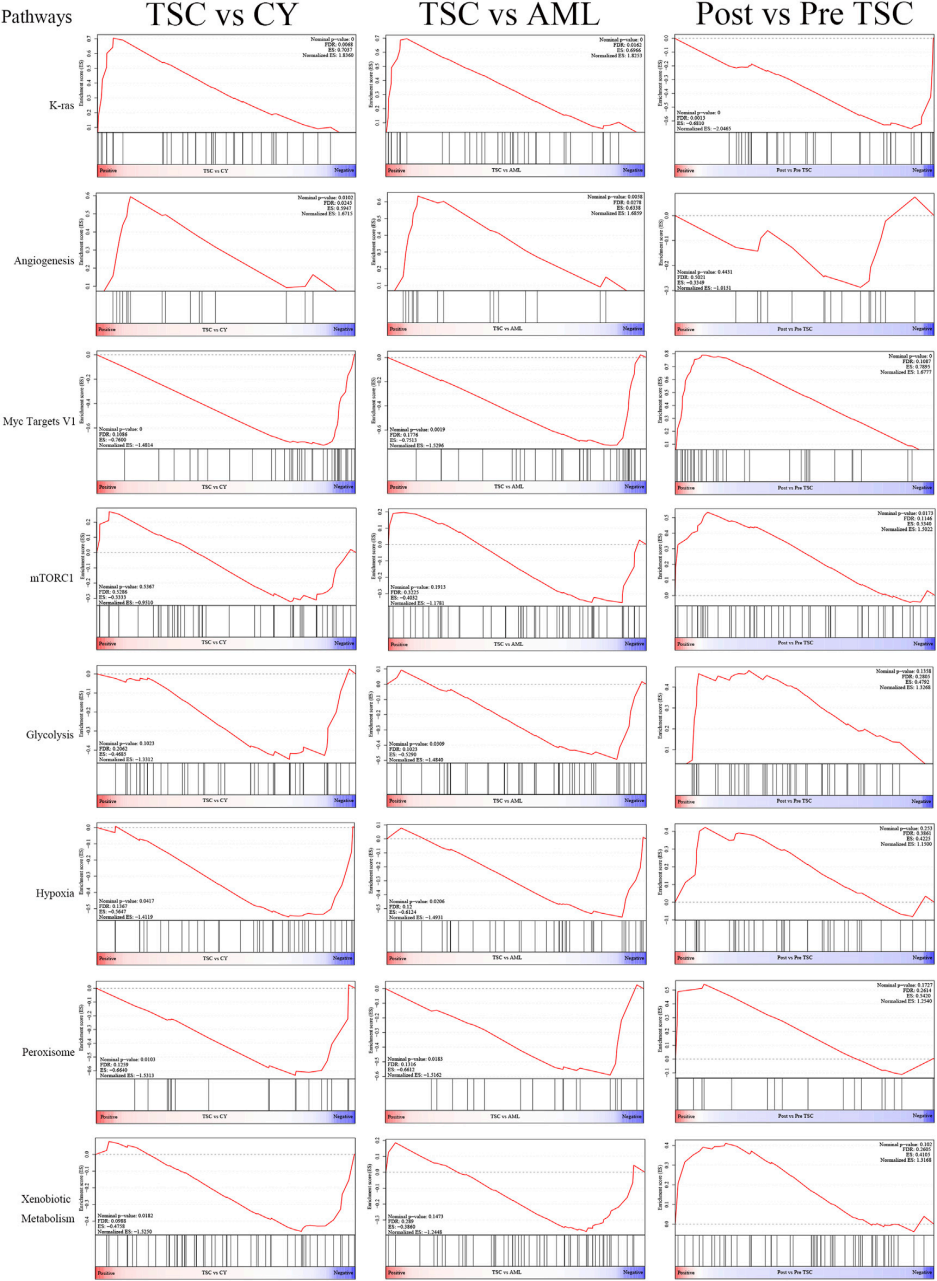
The display of significant GSEA enrichment results within the different subgroups.

Differential analysis was also carried out within the post_TSC versus pre_TSC groups to assess the effect of everolimus on plasma proteomics. With the corresponding cutoff value, 40 upregulated and 28 downregulated molecules were observed (presented in Figure 3A, below), and the GO analysis revealed altered nuclear-transcribed mRNA catabolic process, mRNA catabolic process, and protein targeting to ER pathways after everolimus treatment (Figure 3B, below). The GSEA pathway analysis revealed upregulated MYC targets V1, estrogen response late, interferon gamma response and the mTORC1 signaling pathway. Interestingly, treatment with everolimus reversed almost all of the altered pathways caused by the TSC gene mutations (Figure 4).

### Diagnostic and prognostic role of serum proteomics in TSC-RAML

To find potential biomarkers that could not only distinguish TSC-RAML from renal cysts and S-AML but also predict the response to everolimus, the DE proteins within the different subgroups were analyzed.

Finally, 34 intersecting molecules were observed (Supplementary Figure S2A). The top 11 upregulated and downregulated proteins are shown in Table 2. From the expression pattern, we can clearly see that most upregulated proteins returned to normal levels after everolimus treatment and *vice versa*.

**TABLE 2:** Potential diagnostic and prognostic proteins of TSC-RAML.

Proteins	TSC vs. CY			TSC vs. S-AML			Post vs. Pre TSC		
	FC	*p*-value	AUC	FC	*p*-value	AUC	FC	*p*-value	AUC
PMEL	29.470	7.738*10^−16^	0.98	4.013	8.235*10^−6^	0.80	0.417	0.003	0.72
N4BP2	23.410	8.112*10^−23^	0.96	7.094	4.641*10^−15^	0.93	0.197	1.245*10^−11^	0.88
PCSK1N	19.641	1.319*10^−22^	0.97	8.969	2.364*10^−15^	0.94	0.306	1.287*10^−8^	0.85
AEBP1	18.236	4.138*10^−20^	0.96	19.609	3.524*10^−21^	0.99	0.254	7.560*10^−8^	0.87
TGFBR3	6.789	2.314*10^−12^	0.94	4.141	6.437*10^−9^	0.90	0.395	7.270*10^−7^	0.82
SDHA	6.475	2.174*10^−12^	0.90	2.772	3.488*10^−9^	0.85	0.300	4.956*10^−11^	0.90
CEACAM1	4.274	4.455*10^−11^	0.85	3.148	4.153*10^−10^	0.84	0.479	2.463*10^−5^	0.74
PIGR	3.507	8.261*10^−11^	0.85	2.587	7.037*10^−9^	0.82	0.351	3.544*10^−7^	0.86
COL15A1	3.203	4.183*10^−8^	0.81	3.276	8.893*10^−9^	0.84	0.437	9.088*10^−6^	0.80
PDCD1LG2	3.115	9.367*10^−8^	0.80	2.088	2.152*10^−6^	0.78	0.283	1.221*10^−10^	0.85
SFTPD	2.865	1.106*10^−6^	0.77	3.761	8.652*10^−10^	0.85	0.204	2.319*10^−9^	0.87
GOT2	0.052	1.775*10^−3^	77.1	0.116	9.052*10^−3^	0.76	6.584	0.049	0.65
RPS3	0.054	6.529*10^−18^	0.97	0.079	7.896*10^−11^	0.90	2.662	5.470*10^−6^	0.81
ACP1	0.061	7.662*10^−27^	0.98	0.093	4.018*10^−6^	0.95	4.153	8.919*10^−6^	0.96
HK1	0.071	1.245*10^−13^	0.96	0.473	3.383*10^−4^	0.75	6.133	0.012	0.62
UBA1	0.090	1.889*10^−3^	0.72	0.137	0.036	0.63	6.512	0.049	0.67
NAA15	0.112	9.777*10^−3^	0.76	0.095	0.012	0.73	2.142	1.210*10^−8^	0.86
CALD1	0.154	3.338*10^−12^	0.94	0.109	2.028*10^−10^	0.91	5.864	1.831*10^−8^	0.85
FLOT2	0.171	9.640*10^−6^	0.84	0.0890	1.248*10^−22^	0.96	3.083	0.003	0.78
RPS9	0.204	2.093*10^−9^	0.88	0.434	0.041	0.50	3.710	1.493*10^−7^	0.82
YWHAH	0.216	7.075*10^−10^	0.88	0.277	1.788*10^−9^	0.87	3.033	4.405*10^−9^	0.84
ACAT1	0.217	3.956*10^−10^	0.92	0.262	1.596*10^−9^	0.89	3.101	2.377*10^−7^	0.86

**FIGURE 5:**
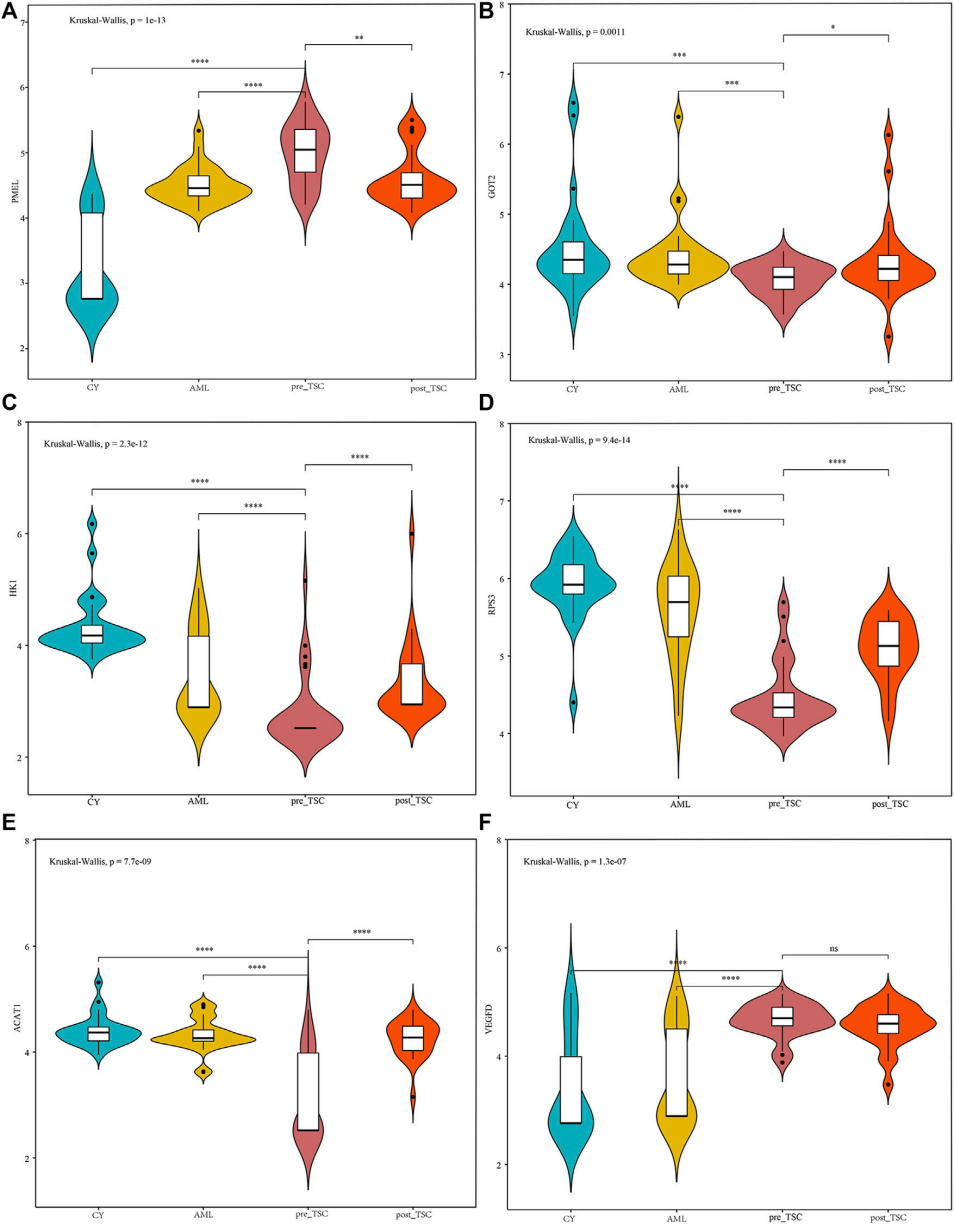
The relative expression level of PMEL **(A)**, GOT2 **(B)**, HK1 **(C)**, RPS3 **(D)**, ACAT1 **(E)** and VEGFD **(F)** within different groups. The above sentence should be added after "Relative proteins levels of some important molecules based on the UPLC-MS results.

From the AUC value, we found that these proteins could perfectly distinguish TSC-RAML from renal cysts and S-AML and within the treatment groups. Furthermore, we compared the correlation between the protein level and maximum tumor volume burden. After applying Pearson analysis, we identified five proteins (out of the 34 intersected DE proteins) positively correlated with the maximum renal angiomyolipoma (*p* < 0.05), namely, PCSK1N, PMEL, HK1, GOT2 and SPTBN2 (as presented in Supplementary Figures 2B–F). Since VEGF-D has been previously proven to be a gold standard biomarker of TSC, we compared the expression level of the intersected proteins with VEGF-D, and many of the proteins demonstrated better discrimination (Figure 5).

### The metabolomics of TSC-RAML, S-AML and renal cysts

To describe the metabolomic profiling of TSC-RAML, S-AML and renal cysts, UPLC-MS was applied to measure the concentrations of small metabolites.

Using the same samples and methods for grouping, we measured the plasma metabolites of 110 samples. After pre-analytical data processing (including quality control, missing value estimation, log transformation and Z score scaling), we identified a total of 517 metabolites for further analysis (Supplementary Figure S1B).

**FIGURE 6:**
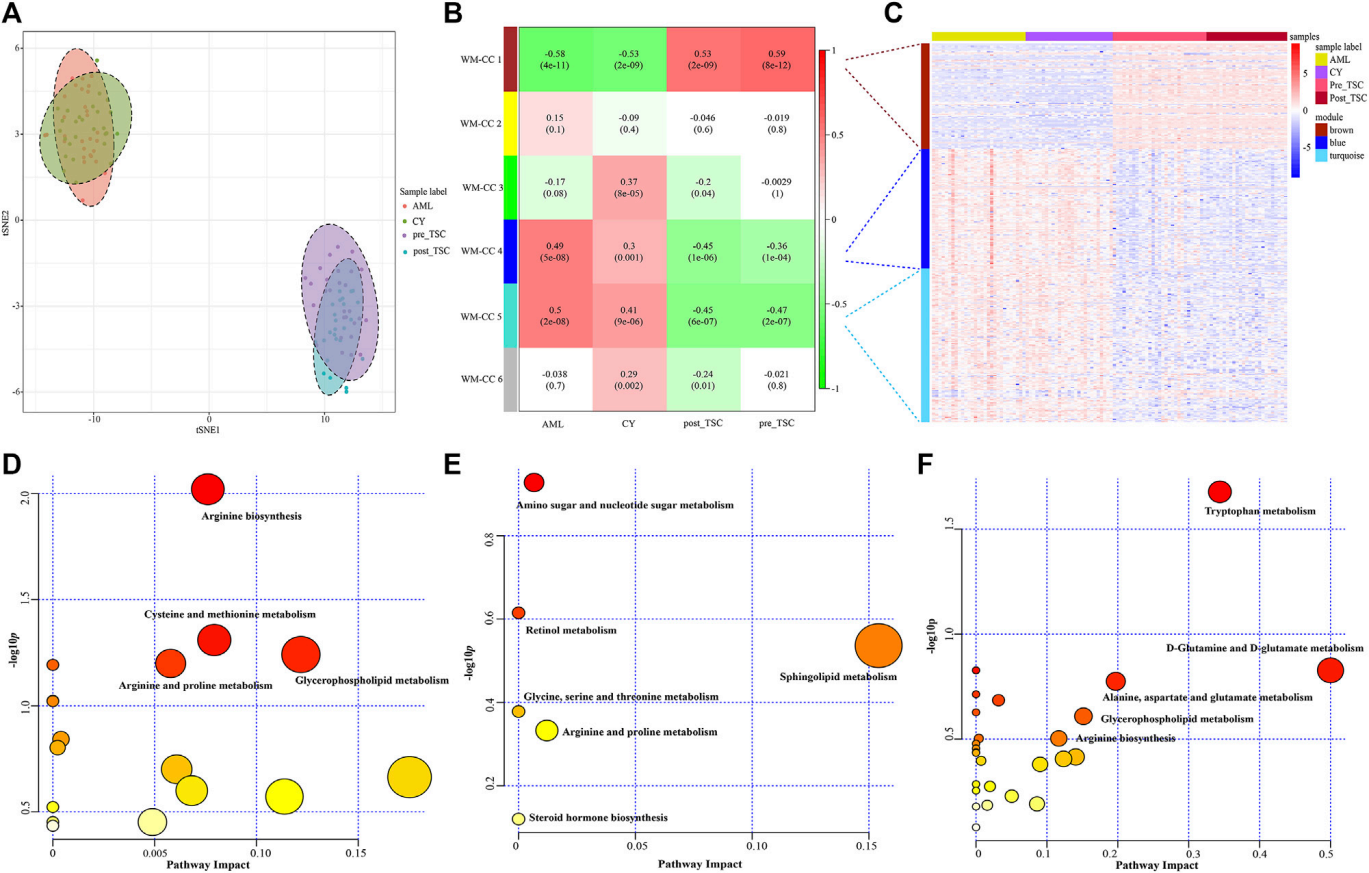
Metabolomics profile of all enrolled patients. **(A)** t-SNE analysis revealed the distinguished metabolomic pattern of TSC-RAML. **(B)** WGCNA identified six whole metabolites co-expression clusters (WM-CCs). **(C)** The relative expression levels of metabolites within three differential modules. **(D)** Pathway analysis based on the “WM-CC 1” module revealed altered arginine biosynthesis. **(E)** Pathway analysis based on the “WM-CC 4” module showed altered amino sugar and nucleotide sugar metabolism. **(F)** Pathway analysis based on the “WM-CC 5” module demonstrated altered tryptophan metabolism.

First, an unsupervised t-SNE analysis (Figure 6A) was launched, and from the results we can clearly see that there was a distinguished altered metabolomic component within the 4 subgroups, especially with the TSC (including pre-treatment and post-treatment TSC-RAML) vs. renal cyst and S-AML, illustrating the specific metabolomic profiling of TSC-RAML.

Similarly, to find the characteristic metabolomic clusters of TSC-RAML, WGCNA was applied and six whole metabolome coexpression clusters (WM-CC) were constructed, within which “WM-CC 1”, “WM-CC 4” and “WM-CC 5” were significantly correlated with TSC-RAML (Figure 6B). The metabolite expression levels of the different modules were obviously different within subgroups (Figure 6C). Furthermore, the pathway enrichment of the three distinguished modules illustrated their altered metabolomic patterns, including upregulated arginine biosynthesis, cysteine and methionine metabolism as well as downregulated amino sugar and nucleotide sugar metabolism and tryptophan metabolism of TSC-RAML (Figures 6D–F).

### The comparison metabolomics of TSC-RAML versus renal cysts and sporadic AML and altered metabolomic profiles after everolimus treatment

**FIGURE 7:**
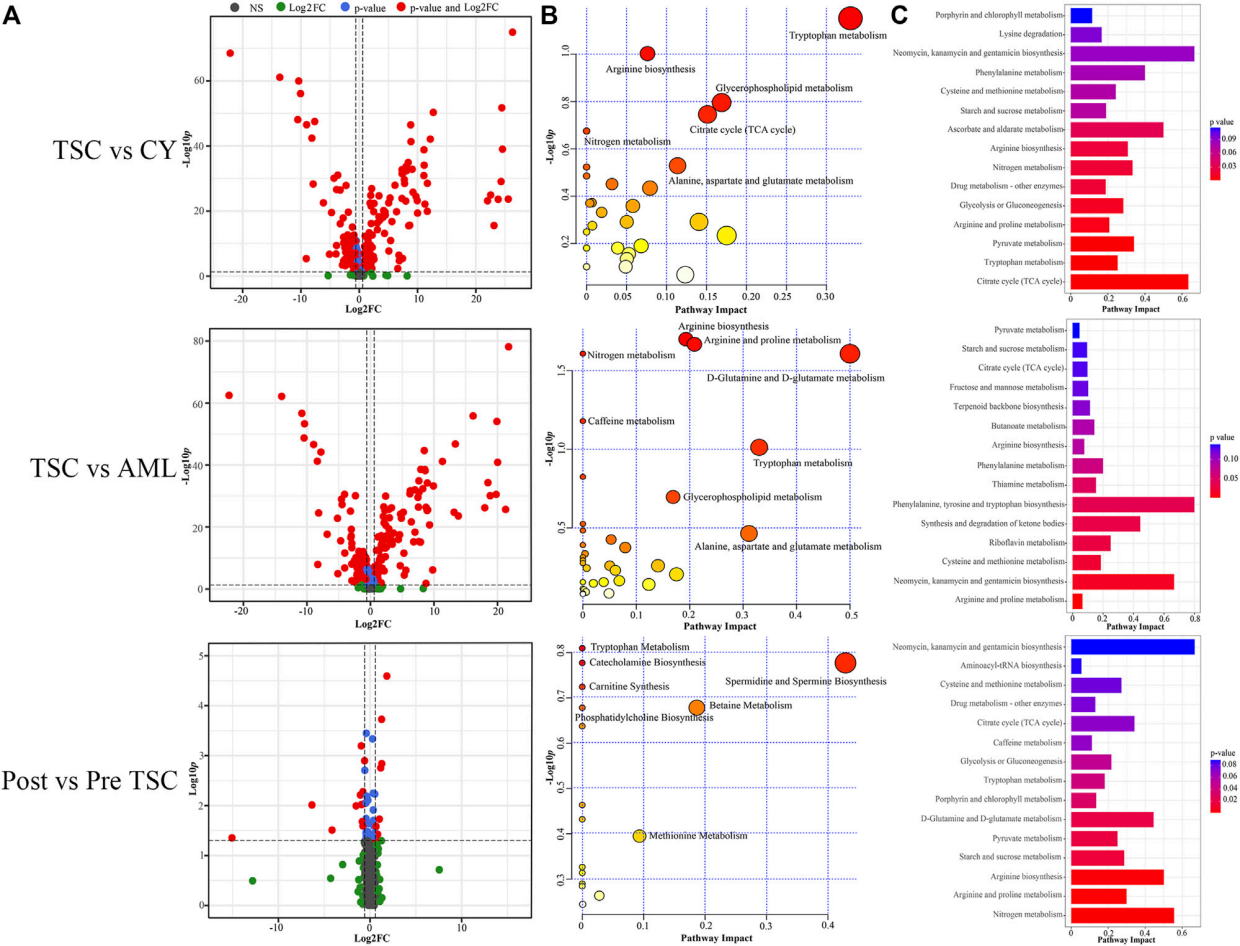
Comparison within different groups and functional analysis of the DE metabolomics. **(A)** Volcano plot based on the threshold. **(B)** Pathway enrichment of DE metabolites showed the many altered metabolomic pattern within different groups, including tryptophan metabolism. **(C)** Joint pathway analysis integrating the DE proteins and metabolites.

Similar to proteomics, the 110 samples were first divided into 4 subgroups (pre-treatment TSC-RAML, post-treatment TSC-RAML, renal cysts and S-AML). When comparing pre-treatment TSC-RAML vs. renal cysts, there were 272 differentially expressed metabolites, namely, 116 upregulated and 156 downregulated metabolomic molecules (depicted as a volcano plot in Figure 7A, above). The pathway analysis revealed dysregulated tryptophan metabolism, arginine biosynthesis and glycerophospholipid metabolism (Figure 7B, above). In addition, the joint pathway that integrates DE proteins and metabolites revealed a critically dysregulated metabolism, including the citrate cycle, tryptophan metabolism and pyruvate metabolomic disturbance (Figure 7C, above).

For the TSC-RAML vs. S-AML group, a total of 283 DE metabolites were confirmed, which included 106 upregulated and 177 downregulated metabolites (as depicted in Figure 7A, middle). The pathway analysis revealed altered D-glutamine and D-glutamate metabolism, nitrogen metabolism and porphyrin and chlorophyll metabolism (Figure 7B, middle). The joint pathway analysis stressed the dysregulated glucose metabolism and nitrogen metabolism (Figure 7C, middle).

Regarding the metabolomic effect of everolimus treatment, 22 DE metabolites were identified for the post-treatment vs. pre-treatment TSC-RAML, including 9 upregulated and 13 downregulated metabolites (Figure 7A, below). The pathway analysis showed that everolimus treatment changed many pathways, including pyrimidine metabolism and tryptophan metabolism (Figure 7B, below). The joint pathway analysis showed many altered amino acid and nucleotide metabolism pathways (Figure 7C, below).

### Potential diagnostic and prognostic metabolite biomarkers of TSC-RAML

**FIGURE 8:**
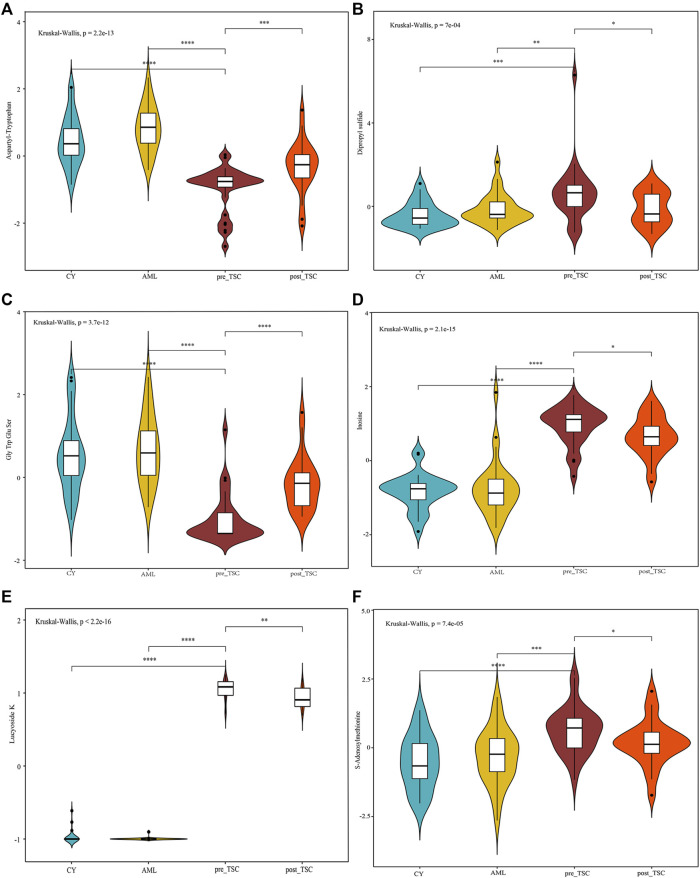
Relative metabolite levels of some important molecules based on the UPLC-MS results. The relative expression level of Aspartyl-Tryptophan **(A)**, Dipropyl sulfide **(B)**, Gly Trp Glu Ser **(C)**, Inosine **(D)**, Lucyoside K **(E)** and S-Adenosylmethionine **(F)** within different groups.

To discover both prognostic and diagnostic metabolites, we chose the intersected DE metabolites within different groups. As a result, 13 DE metabolites were selected (Supplementary Figure S3A), and the corresponding data are presented in Table 3. After assessing the 13 metabolite levels with the maximum tumor volume with Pearson correlation analysis, we did not find any metabolites associated with the maximum tumor volume burden (Supplementary Figures 3B–F). The relative expression levels of some critical metabolites are depicted in Figure 8, from which we can clearly see that treatment with everolimus could reverse the altered metabolite levels caused by the TSC mutations.

**TABLE 3:** Potential diagnostic and prognostic metabolites of TSC-RAML.

Metabolites	TSC vs. CY			TSC vs. S-AML			Post vs. Pre TSC		
	FC	*p*-value	AUC	FC	*p*-value	AUC	FC	*p*-value	*AUC*
Lucyoside K	6,657.429	4.883*10^−51^	1.0	73,025.948	1.443*10^−56^	1.0	0.662	1.26*10^−3^	0.76
His Trp	2,171.442	1.311 *10^−31^	1.0	36.0719	1.762*10^−25^	0.98	1.507	0.0483	0.68
Pro Pro Glu Phe	404.041	2.352*10^−16^	1.0	111.556	5.911*10^−16^	0.99	1.783	0.0377	0.67
Inosine	16.936	8.377*10^−19^	0.98	5.760	2.746*10^−14^	0.95	0.635	0.0240	0.67
Dipropyl sulfide	3.947	5.690*10^−4^	0.79	4.141	9.994*10^−3^	0.73	0.3487	0.0101	0.72
S-Adenosylmethionine	3.037	2.174*10^−12^	0.81	2.236	3.155*10^−4^	0.76	0.581	0.0256	0.71
Gly Trp Glu Ser	0.0671	3.920*10^−10^	0.94	0.0608	2.444*10^−12^	0.95	3.569	2.549*10^−5^	0.87
Adenosine 3′-monophosphate	0.105	8.144*10^−17^	0.96	0.0389	2.938*10^−16^	0.95	0.0565	0.0310	0.57
3,4-Methylenedioxymethamphetamine (MDMA)	0.1478	1.383*10^−18^	1.0	0.123	5.726*10^−20^	1.0	0.481	0.00613	0.71
Gly Asp Ala Ala	0.156	6.092*10^−11^	0.96	0.131	1.910*10^−15^	0.97	3.569	2.549*10^−5^	0.76
Aspartyl-Tryptophan	0.198	8.160*10^−10^	0.94	0.131	4.811*10^−14^	0.99	2.429	0.00145	0.79
Ketotifen-N-glucuronide	0.265	8.674*10^−6^	0.83	0.363	6.097*10^−4^	0.76	0.5145	6.686*10^−4^	0.75
alpha-Terpineol formate	0.547	1.776*10^−8^	0.91	0.524	6.846*10^−7^	0.87	0.6539	0.00913	0.70

## Discussion

In summary, our proteomics analysis found an upregulated angiogenesis pathway, while metabolomics showed the multiple altered amino acid pathways, such as the arginine biosynthesis, tryptophan metabolism and glutamate metabolism. In addition, plasma proteins such as PMEL and metabolites such as S-adenosylmethionine showed potential diagnostic and prognostic functions, demonstrating a significant role in translational medicine, which fills a knowledge gap in this field.

### Functional analysis of proteomics

From the GSEA functional enrichment of TSC-RAML, we found that the angiogenesis pathway was significantly upregulated compared with both renal cysts and sporadic AML patients. The WGCNA cluster and ClueGO enrichment also identified characteristic upregulation of smooth muscle cell migration and proliferation in TSC-RAML patients. As the name suggests, angiomyolipoma is comprised of different proportions of proliferative blood vessels, smooth muscle and adipose tissues (Lam et al., 2018). Arbiser, J. L. et al. proved that TSC-associated benign neoplasms, including renal angiomyolipoma, are highly vascular and possess the ability to synthesize and secrete VEGF *in vitro* (Arbiser et al., 2002). Later, researchers found that mTOR1 plays a central role in the process of angiogenesis through multifactorial ways, including promoting VEGF-A expression by HIF-1α dependent and HIF-1α independent mechanism (Dodd et al., 2015). Based on this hypothesis, additional experiments have suggested that a combination of rapalogs (Rapamycin and its analogs) and angiogenesis inhibitors, such as everolimus plus sorafenib, may significantly decrease the tumor size and improve the therapeutic efficacy by inhibiting mTORC1 and the mitogen-activated protein kinase (MAPK) pathway (Yang et al., 2017), which is superior to the treatment with single rapalogs alone. Another study also found that angiogenesis inhibitors (sunitinib and bevacizumab) have therapeutic effects on TSC-related tumors, although they are not as effective as rapamycin (Woodrum et al., 2010).

Another significantly upregulated pathway relative to renal cysts and sporadic AML was K-RAS pathway activation, which has been proven to play a critical role in the tumorigenesis of various cancers and therefore has been implicated as a cancer target during the past few years, such as in pancreatic ductal adenocarcinoma (Mehra et al., 2021), lung cancer (Chu, 2020), and breast cancer (Gupta et al., 2020). As an important downstream target of the K-RAS signaling pathway, the role of PI3K-Akt-mTOR axis in tumor occurrence and development has been validated by a variety of researchers (Hillmann and Fabbro, 2019). Although many drugs targeting the K-RAS pathway have been explored to induce tumor regression in other diseases (Kinross et al., 2011; Hillmann and Fabbro, 2019), the evidence for their use in TSC-RAML is limited. Therefore, our results may provide new ideas for the treatment of rapamycin-resistant TSC-RAML.

### Protein biomarkers for differential diagnosis and everolimus effect prognostication

In our analysis, we discovered that the plasma pre-melanosome protein PMEL, antigen for HMB-45, demonstrates good differential (AUC of TSC vs. CY: 0.98; AUC of TSC vs. AML: 0.80) and prognostic ability (AUC of Post vs. Pre TSC: 0.72), as depicted in Table 2. In addition, the PMEL level was also associated with the tumor burden (r = 0.55, *p* < 0.001, as depicted in Figure Supplementary Figure S2E). To the best of our knowledge, our study is the first to discover the latent role of plasma PMEL in diagnosing and predicting the outcome of TSC-RAML. Pigment cell-specific PMEL is an extraordinarily well-conserved type I transmembrane glycoprotein mainly engaged in the formation of fibrillar sheets within melanosomes (Watt et al., 2013), and it is associated with melanocyte-related diseases and pathological neurodegeneration, such as Alzheimer’s Disease (AD) and Parkinson’s disease (PD) (Watt et al., 2013).

In 2001, Stone, C. H. assessed the relationship between the immunophenotypic and ultrastructural profile of renal angiomyolipoma and found that all 27 renal angiomyolipomas stained positive for HMB-45, regardless of their identification as epithelioid, spindle, or adipocytic cells, suggesting all components were coming from a common cell ancestor and providing a unitarian concept for renal angiomyolipoma (Stone et al., 2001). In addition to angiomyolipoma, pulmonary LAM cells are also positive for HMB-45 (Venyo, 2016; Guo et al., 2020), indicating that neural crest cells, a kind of migratory, multipotent embryonic cell, maybe the cell origin for LAM and other TSC-related tumors (Delaney et al., 2014). Two recent published articles have found the relative reduction of T lymphocytes within the tumor microenvironment for TSC related LAM (Guo et al., 2020) and AML (Wang et al., 2022b), suggesting adoptive transferred PMEL-specific CD8^+^ T cells may be effective because this cytotoxic T cells can specifically attack PMEL + tumor cells (Hanada et al., 2019; Han et al., 2020).

Another protein, PCSK1N, also called proSAAS, an inhibitor of prohormone convertase 1 (PC1) activity produced by neuroendocrine cells, has been proven to be a biomarker for many neurological disorders, including Alzheimer’s disease (AD), Pick’s disease, and the Parkinsonism-dementia complex (Shakya et al., 2020; van Steenoven et al., 2020). Encoded by the *PSCK1N* gene, ProSAAS was initially identified as a neuroendocrine-specific proprotein convertase binding protein and was classified into the granin family of proteins (Shakya et al., 2020). In addition, proSAAS can be proteolytically processed into a large number of active neuropeptides, including SAAS, PEN and LEN, all of which have been regarded as neurotransmitters (Khoonsari et al., 2019). Several proteomic and transcript studies have found elevated proSAAS protein levels in cerebrospinal fluid and upregulated proSAAS expression in the brain during Alzheimer’s progression (McDermott et al., 2019).

More than 90% of TSC patients have central nervous system abnormalities, including cortical or subcortical tubers, subependymal nodules, giant cell astrocytoma, and white matter migration lines (Curatolo et al., 2015). These pathological lesions can lead to many neurological symptoms, such as epilepsy and tuberous sclerosis-associated neuropsychiatric disorders (TANDs). In our analysis, we found that plasma PCSK1N was significantly elevated compared with renal cyst (FC = 19.6, *p* = 1.3*10^−22^) and S-AML (FC = 8.97, *p* = 2.36*10^−5^) but was reduced dramatically after everolimus treatment (FC of post vs. pre = 0.3, *p* = 1.29*10^−8^), which indicated that plasma PCSK1N may be a useful marker for TSC.

Furthermore, some other biomarkers, including SDHA, GOT2, HK1 and ACAT1, are involved in metabolic processes, including glucose metabolism, and amino acid and fatty acid metabolism, indicating the wide reprogramming of vital metabolic and biochemical processes caused by *TSC* gene mutations (Lam et al., 2018).

### Functional analysis of metabolomics

In our comparative metabolomic analysis, we also found characteristic plasma metabolomic patterns of TSC-RAML, including arginine biosynthesis, glutamine and glutamate metabolism, tryptophan metabolism, and glycerophospholipid metabolism, which was consistent with the joint pathway analysis integrating proteomics and metabolomics (as depicted in Figures 6, 7). The overactivated mTOR pathway caused by dysfunctional hamartin or tuberin could lead to a subsequent metabolic alteration to sustain necessary proliferation and survival, including aberrant metabolism of amino acids, glucose, nucleotides, fatty acids and lipids. On the other hand, the altered metabolites, particularly amino acids such as arginine and glutamine (Wolfson and Sabatini, 2017), could reversely stimulate mTOR *via* RAS related GTP binding proteins (Mossmann et al., 2018), resulting in positive feedback. As one of the several amino acids that can directly activate the mTOR pathway, arginine can modulate cellular signaling pathways through many mechanisms, such as been transformed into the cytoplasm by solute carriers (SLCs) or by binding to L-amino acid receptor, G-protein coupled receptor GPRCA6 (Chen et al., 2021). In contrast, deprivation of arginine could convert Rag GTPases into an inactive state and lead to the immediate deactivation of mTORC1 (Darnell et al., 2018), thus suppressing the growth and inducing cell death of various cancer types, and corresponding clinical trials are being conducted (Chen et al., 2021). Our metabolomic analysis showed that arginine biosynthesis was significantly upregulated and that the fold change in L-arginine could even reach 2.183 and 1.89 compared with renal cysts and AML, respectively (*p* < 0.01), which suggested that arginine-targeted drugs or an arginine-light diet may be a promising choice for TSC-RAML patients.

In contrast to arginine directly activating the mTOR pathway, glutamine can activate mTOR through a Rag GTPase-independent pathway and it requires the participation of ADP-ribosylation factor 1 (Arf1) (Yan et al., 2020). In addition, TSC-deficient cells have also demonstrated increasing consumption of glutamine to engage in an overactive tricarboxylic acid cycle (which has already been depicted in Figure 7) and create the antioxidant agent glutathione (Lam et al., 2018). Another important pathway, glycerophospholipid metabolism, which has been reported by Bottolo, L. et al. in their research regarding TSC-related LAM, was associated with the severity of lung disease and total body burden of LAM (Bottolo et al., 2020). In our study, however, glycerophospholipids showed an upregulated tendency but it did not reach statistical significance (*p* > 0.05). We think the difference may be due to the inner heterogeneity with TSC-LAM and TSC-RAML and the limited samples within our two studies. Therefore, larger sample size and more centers should be engaged to validate these results.

### Metabolite biomarkers for differential diagnosis and prognosis prediction

Regarding prognostic and diagnostic biomarkers, several metabolites attracted our attention, including S-adenosylmethionine, inosine, and adenosine 3′-monophosphate. As one of the most important methyl donors, S-adenosylmethionine (SAM) plays a critical role in the methylation of multiple biological processes, including DNA, RNA and histone methylation as well as the synthesis of creatine and phosphatidylcholine (Elango, 2020; Menezo et al., 2020), which may be the reason why the level of inosine showed the same tendency as SAM (as depicted in Figure 8). Researchers have found that intracellular SAM can be detected by SAMTOR, a sensor for SAM binding with KICTOR, thus leading to mTORC1 activation and autophagy suppression (Kitada et al., 2021). In addition, SAM is also the sole donor of aminopropyl groups, which have been proven to be overexpressed in various cancers and are vital for cell proliferation (Kaiser, 2020). SAM mainly originates from methionine and ATP under the catalysis of methionine adenosyltransferase (MAT). MAT contains three isozymes in mammals. MAT1 and MAT3 are limited in hepatocytes, while MAT2 are widely expressed in almost all tissues (Alam et al., 2022). Accumulating evidence suggested that SAM and its enzyme MAT2A are closed related with tumorigenesis of various cancers, like colon and breast cancers (Alam et al., 2022). Targeting SAM or MAT2A has proven beneficial among several type of cancers, especially in *MTAP*-deleted cancers (Bruce et al., 2021; Kalev et al., 2021). We suggest that a high level of plasma SAM could satisfy a higher demand for nutritional supplies and altered methylation pattern to sustain tumor progression, indicating that SAM could be a potential pharmacological target, and further research is required.

As retrospective research, our study has some potential disadvantages. First, the small sample size due to the essence of rare disease and lack of external validation may limit the wide application of biomarkers in clinical. To overcome this drawback, we are carrying out multi-center cooperation and the result will be published once finished. In addition, although we have discovered many biomarkers for TSC-RAML, more vitro and vivo experiments targeting the molecules should be carried out to explore the inner mechanism.

**FIGURE 9:**
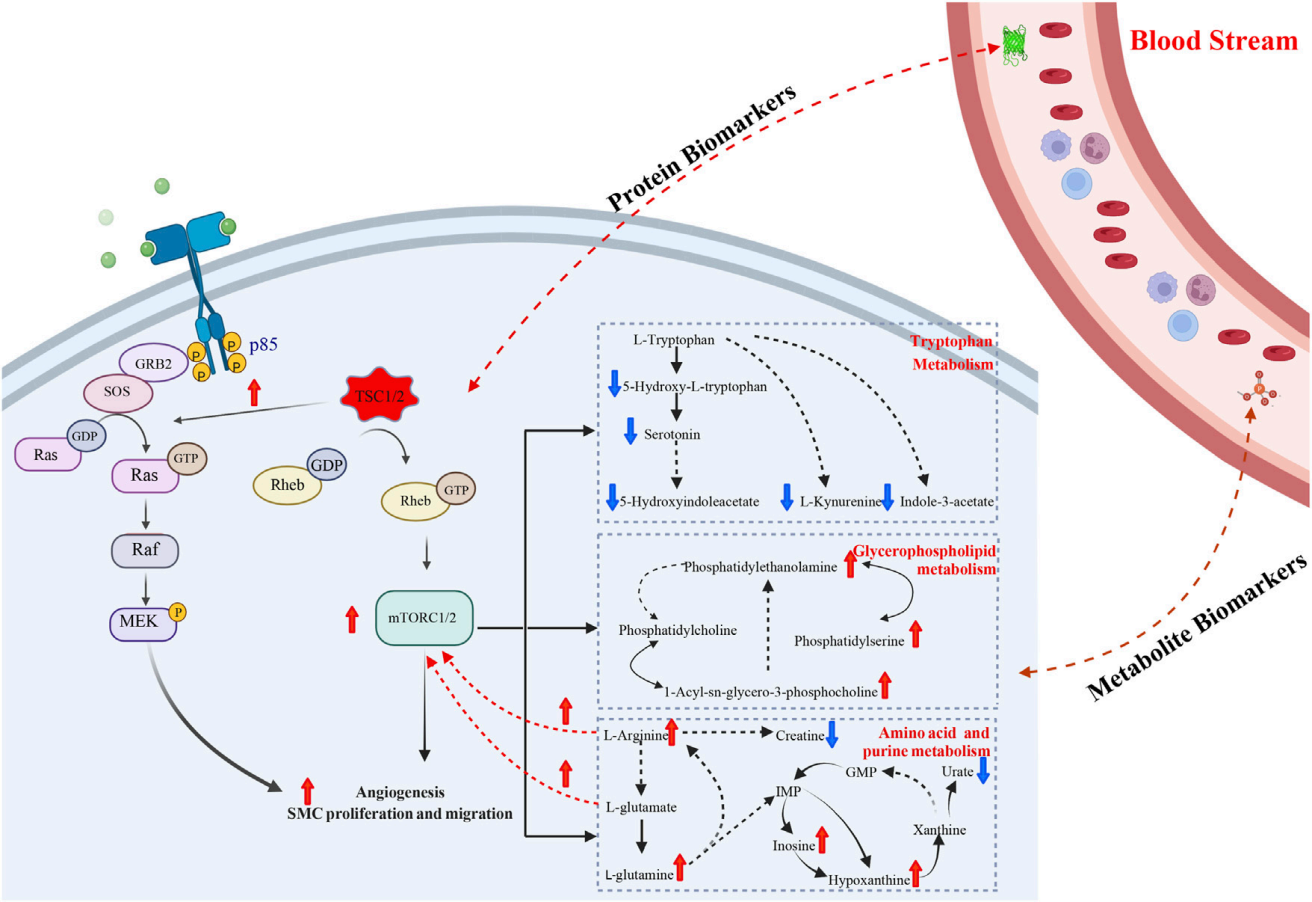
The main summary of discovery in our multi-omics analysis.

In conclusion, we integrated the plasma proteomics and metabolomics data of TSC-RAML and discovered altered unique pathways as well as potential prognostic and diagnostic biomarkers (as summarized in Figure 9). Our results provide new thoughts regarding the underlying mechanism of TSC-RAML and potential drug targets for future research.

## Data Availability

The data presented in the study are deposited in the Genome Sequence Archive (https://ngdc.cncb.ac.cn/bioproject/) under the accession number 'PRJCA014958'.

## Abbreviations

AML, angiomyolipoma; CY, renal cyst; DE, differentially expressed; GO, gene oncology; GSEA, gene set enrichment analysis; LAM, lymphangioleiomyomatosis; mTOR, mammalian target of rapamycin; PMEL, pre-melanosome protein; QC, Quality control; RA, rhabdomyosarcoma; SAM, S-Adenosylmethionine; S-AML, sporadic angiomyolipoma; SEGA, subependymal giant cell astrocytoma; TSC, Tuberous sclerosis complex; TSC-RAML, TSC related AML; t-SNE, t-Distributed Stochastic Neighbor Embedding; UPLC-MS, ultra-performance liquid chromatography-mass spectrometer; WGCNA, weighted gene correlation network analysis; WM-CC, whole metabolome co-expression clusters; WP-CC, whole proteome co-expression clusters.
