# Supplementary material for: UPLC-MS based integrated plasma proteomic and metabolomic profiling of TSC-RAML and its relationship with everolimus treatment
Source: Front Mol Biosci. 2023 Feb 20;10:1000248. doi: 10.3389/fmolb.2023.1000248 (PMC9986496; doi:10.3389/fmolb.2023.1000248)
Supplement: Supplementary file 1 [file DataSheet1.PDF]

Supplementary Table 1. The TSC gene mutations of all enrolled TSC patients

samples	detected mutation	location	Pathologenic or not	Significance
A1	c.5024C>T	TSC2	Pathogenic	Missense mutation, p.Pro1675Leu
C4	c.5126 C>T	TSC2	Pathogenic	Missense mutation,p.Pro1709Leu
E3	c.5228G>A	TSC2	Pathogenic	Missense mutation, p.Arg1743Gln
E9	NM_000548.4:c.1831C>T	TSC2	Pathogenic	Missense mutation, p.Arg611Trp
A2	NM_000548.4:c.3685C>T	TSC2	Pathogenic	Nonsense mutation, p.Gln1229*
A3	NM_000548.4:c.2194C>T	TSC2	Pathogenic	Nonsense mutation, p.Gln732*
A4	Negative	Negative	Negative	Negative
A5	NM_000548.4:c.1874C>G	TSC2	Pathogenic	Nonsense mutation, p.Ser625*
A6	NM_000548.4:c.2194C>T	TSC2	Pathogenic	Nonsense mutation, p.Gln732*
A7	Negative	Negative	Negative	Negative
A8	Negative	Negative	Negative	Negative
A9	Negative	Negative	Negative	Negative
C1	NM_000548.4:c.1047dup	TSC2	Pathogenic	Frameshift mutation, p.Arg350*
C2	NM_000548.4:c.2590C>T	TSC2	Pathogenic	Nonsense mutation, p.Gln864*
C3	NM_000548.4:c.5027_5068+32del	TSC2	Likely pathogenic	Base deletion on exon 39,p.Leu1676_Asp1690delinsHis
C5	c.1947-1G>C	TSC2	Likely pathogenic	Intron, splice variation, c.1947-1G>C
C6	NM_000548.4:c.2233_2234del	TSC2	Pathogenic	Frameshift mutation, p.Lys745Aspfs*16
C7	NM_000548.4:c.208dup	TSC2	Pathogenic	Frameshift mutation, p.Thr70Asnfs*5
C8	c.203_204 insA	TSC2	Likely pathogenic	c.203_204 insA,p.Ala68AlafsX7
C9	c.4255C>T	TSC2	Pathogenic	Nonsense mutation, p.Gln1419Ter
E1	c.788_789insC	TSC2	Likely pathogenic	Frameshift mutation, p.Leu263LeufsX75
E2	NM_000548.4:c.1513C>T	TSC2	Pathogenic	Nonsense mutation, p.Arg505*
E4	Negative	Negative	Negative	Negative
E5	EX22_24 DEL	TSC2	Likely pathogenic	deletion, EX22_24/CDS21_23
E6	c.3683_3684insG	TSC2	Likely pathogenic	Frameshift mutation, p.Leu1228LeufsX6
E7	c.3601_3602insGGCCC	TSC2	Likely pathogenic	Frameshift mutation, p.Thr1203GlyfsX9
E8	c.1507C>T	TSC2	Likely pathogenic	Nonsense mutation, p.Gln503Ter
F8	Negative	Negative	Negative	Negative
F9	NM_000548.4:c.1513C>T	TSC2	Pathogenic	Nonsense mutation

Lable	represent	count
	Nonsense mutation	9
	Negative	6
	Frameshift mutation	6
	Missense mutation	4
	Others	4

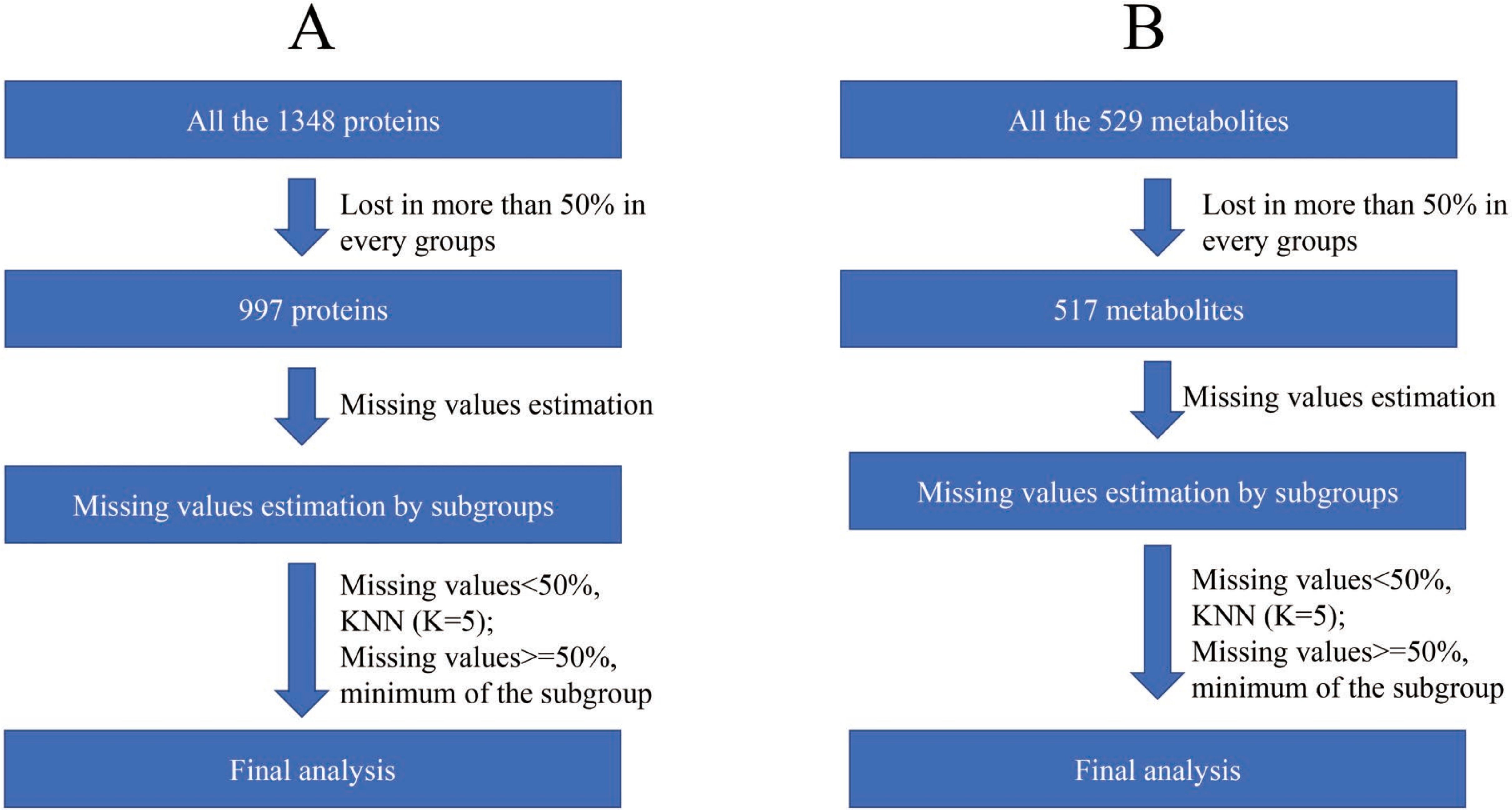

Supplementary figure 1. The pre-analytic data processing procedures of proteomics (A) and metabolomics(B).

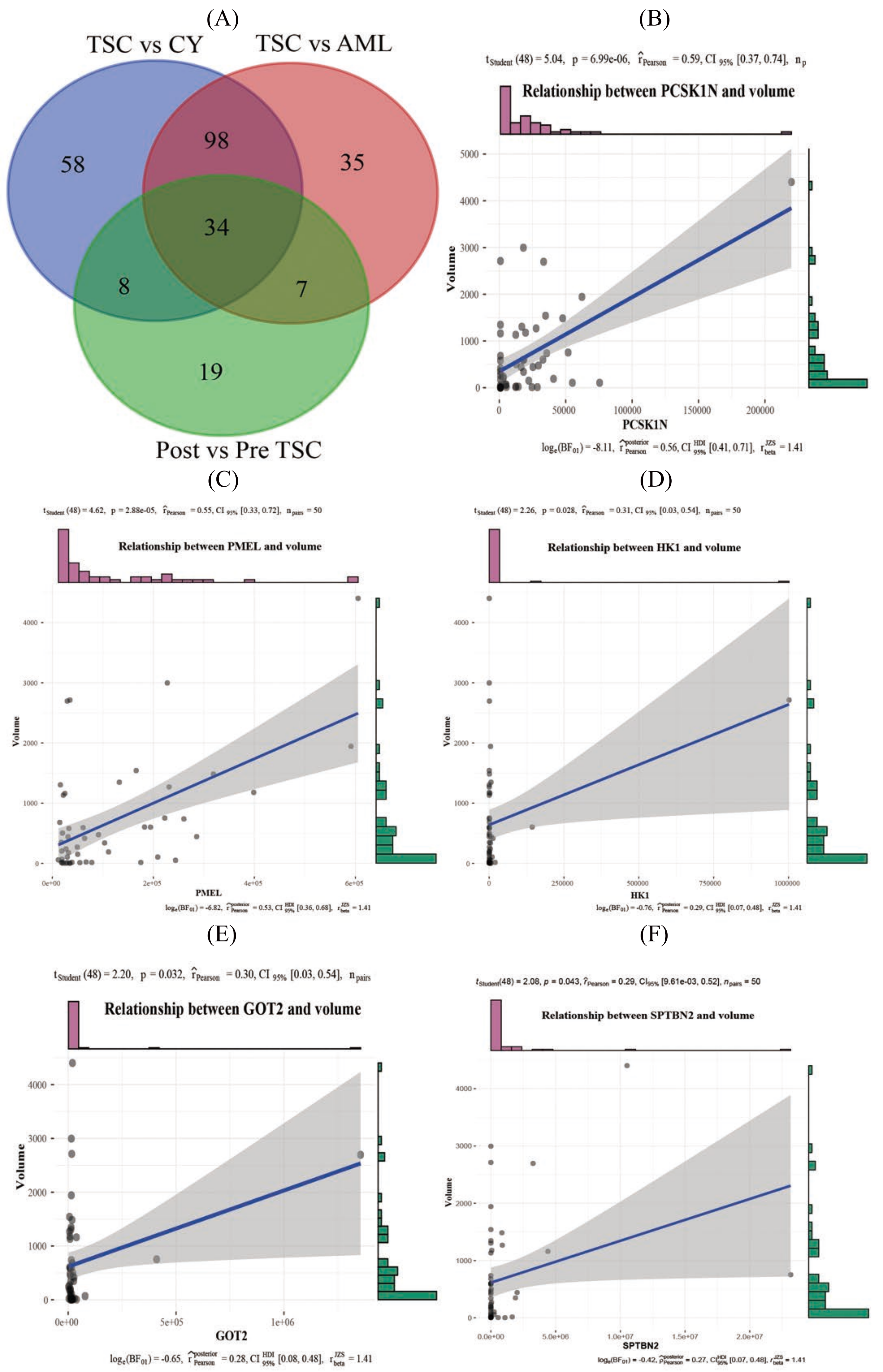

Supplementary figure 2. The intersected differentially expressed proteins (A). The relationship of protein level with maximum tumor volume (B-F).

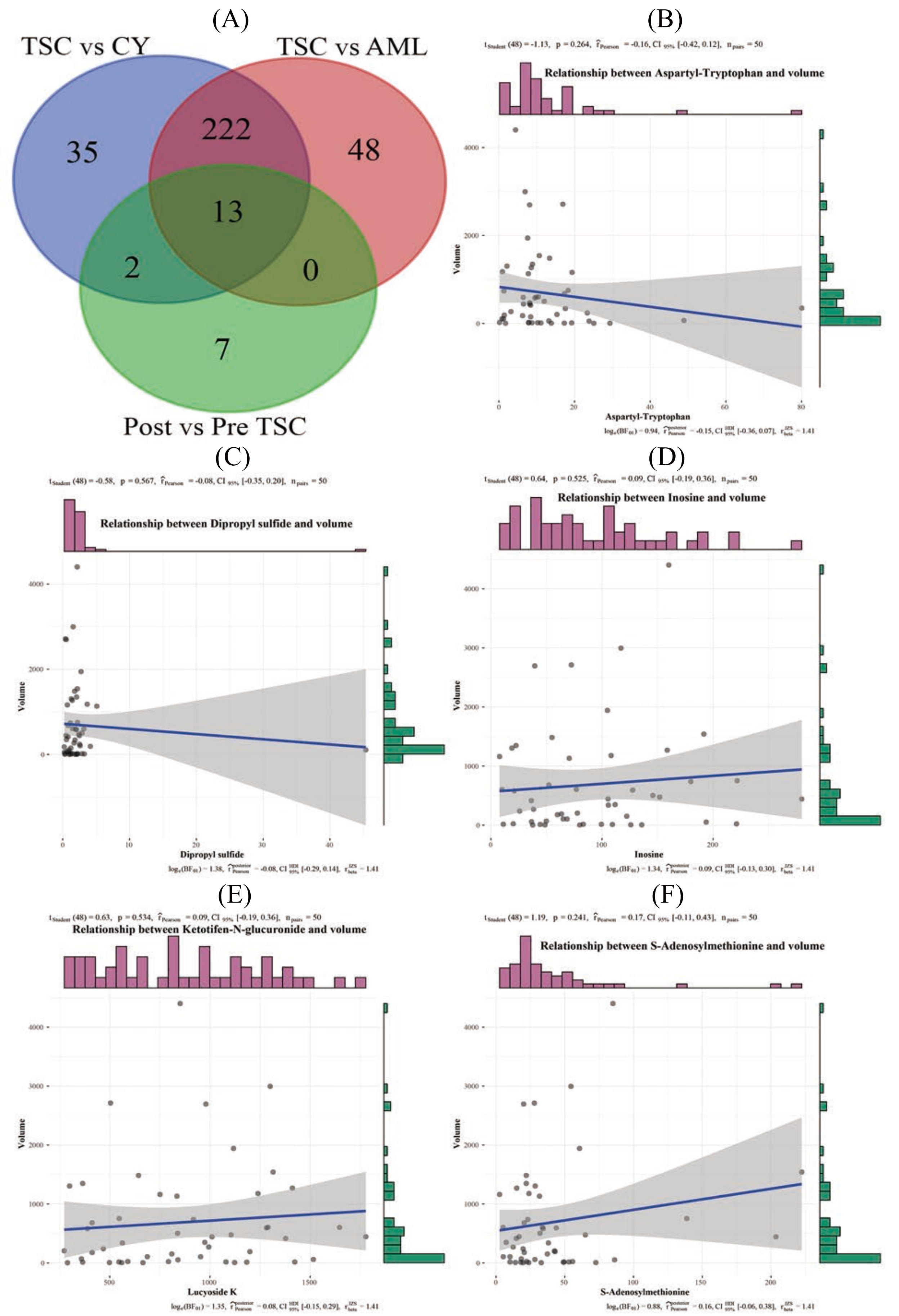

Supplementary figure 3. The intersected differentially expressed metabolites (A). The relationship of metabolite level with maximum tumor volume (B-F).

Supplemental Materials

Supplementary Materials 1: high abundant proteins depletion process.

The directions of High Select™ Top14 Abundant Protein Depletion Mini Spin Columns (Thermo Fisher Scientific, MA. USA). The procedures could be simplified as follows:

(1). Take the columns out from the 4 °C refrigerator and equilibrate the depletion spin column to room temperature.

(2).Remove the column screw cap and add up to 10 μL of sample directly to the resin slurry in the column.

(3).Cap the column and invert the column several times until the resin is completely homogenous in solution.

(4).Incubate the mixture in the column with gentle end-over-end mixing for 10 minutes at room temperature. Make sure the sample mixes with the resin during incubation period.

(5).After incubation, snap off the boĴom closure and loosen the top cap. Place the mini column into a 2 mL collection tube and centrifuge at 1,000 × g for 2 minutes.

(6).Discard the column containing the resin.

(7).Filtrate contains sample with albumin, IgG, and other abundant proteins removed. Use for further processing or store at -20°C for later use. The depleted sample will be in 10 mM PBS and 0.02% sodium azide, pH 7.4.

(8).After that, we could get 300 ul plasma with the high-abundant proteins removed. Take 10 ul from each sample to measure the protein concentration by BCA assay (Pierce).

Supplemental Material 2

2.1 Data processing using Progenesis QI

The detailed workflow for data processing facilitated by Progenesis QI is involved "create a new experiment", "import data", "review alignment", "experiment design setup", "peak picking", "reviewed convolution--normalization", and "identify compounds" in sequence. In general, the whole process ran automatically using optimized parameter settings. (1) In the

stage of create a new experiment, adduct ion was carefully selected as it would influence the number of characterized compounds and also the identification accuracy. Based on the ionization behaviors of reference standards, the adduct ion forms, comprising [M + H]+, [M + Na]+, [M + K], [M + NH4]+, [2M + H]+, [2M + Na]+,[2M + NH4]+, [M + H – H2O]+ and [M + H – 2H2O]+ , were selected. (2) The MS data acquired by LC-MS for all the URINE samples were imported into the Progenesis QI software, generating a 2D ion intensity map with the retention time and m/z information as the ordinate and abscissa, respectively. (3) Peak alignment was carried out in automatic manner taking a QC run as the reference, the score values for all the samples were greater than 90 %. (4) For peak picking, the thresholds of chromatographic peak absolute intensity, and retention time limits can be set to achieve the maximum real ion signals with noise excluded. In the present study, absolute intensity and retention time limit were set at 1000 and default. "Normalize to all compound" was used to normalized peaks to eliminate sampling and analysis bias. (5) Further compound identification was performed by searching the HMDB database (2017 version). The identification results combined with the intensity data were exported as .csv files for subsequent compound confirmation and multivariate statistical analysis.

2.2 Confirmation of compounds characterization

Detailed compound identification information (.csv file) included compound ID, adducts, formula, score, fragmentation score, mass error (in ppm), isotope similarity, theoretical isotope distribution, web link, and m/z values. The data was further analyzed in detail, under which more abundant MS/MS fragments were acquired. Confirmation of the differential compounds was performed by the parameters, including Score, Fragmentation score, and

Isotope similarity given by Progenesis QI. Score ranging from 0 to 60, is used to quantify the reliability of each identity. According to the score results of the reference standards, the threshold was set at 35.0. Fragmentation score represents the matching degree between the theoretical fragments and the measured ones. The fragmentation score of 0 indicates no match occurs or the compound generates no fragments. Isotope similarity is calculated by comparison of the measured isotope distribution of a precursor ion with the theoretical. The compound identification is more reliable the higher the values obtained.
